# Nonaggressive behavior: A strategy employed by an obligate nest invader to avoid conflict with its host species

**DOI:** 10.1002/ece3.6572

**Published:** 2020-07-24

**Authors:** Helder Hugo, Paulo F. Cristaldo, Og DeSouza

**Affiliations:** ^1^ Centre for the Advanced Study of Collective Behaviour University of Konstanz Konstanz Germany; ^2^ Department of Collective Behaviour Max Planck Institute of Animal Behavior Radolfzell Germany; ^3^ Department of Biology University of Konstanz Konstanz Germany; ^4^ Lab of Termitology Federal University of Viçosa Viçosa Brazil; ^5^ Department of Agronomy Federal Rural University of Pernambuco Recife Brazil

**Keywords:** Aggressiveness, Cohabitation, *Constrictotermes cyphergaster*, Inquilinism, *Inquilinitermes microcerus*, Symbiosis

## Abstract

In addition to its builders, termite nests are known to house a variety of secondary opportunistic termite species so‐called inquilines, but little is known about the mechanisms governing the maintenance of these symbioses. In a single nest, host and inquiline colonies are likely to engage in conflict due to nestmate discrimination, and an intriguing question is how both species cope with each other in the long term. Evasive behaviour has been suggested as one of the mechanisms reducing the frequency of host‐inquiline encounters, yet, the confinement imposed by the nests' physical boundaries suggests that cohabiting species would eventually come across each other. Under these circumstances, it is plausible that inquilines would be required to behave accordingly to secure their housing. Here, we show that once inevitably exposed to hosts individuals, inquilines exhibit nonthreatening behaviours, displaying hence a less threatening profile and preventing conflict escalation with their hosts. By exploring the behavioural dynamics of the encounter between both cohabitants, we find empirical evidence for a lack of aggressiveness by inquilines towards their hosts. Such a nonaggressive behaviour, somewhat uncommon among termites, is characterised by evasive manoeuvres that include reversing direction, bypassing and a defensive mechanism using defecation to repel the host. The behavioural adaptations we describe may play an important role in the stability of cohabitations between host and inquiline termite species: by preventing conflict escalation, inquilines may improve considerably their chances of establishing a stable cohabitation with their hosts.

## Graphical abstract

We investigate the behavioral dynamics of the encounter between an obligate inquiline termite and its host termite species. Hosts are aggressive toward inquilines. When threatened or even physically injured by hosts, inquilines react with low aggressiveness, exhibiting a repertoire of evasive manoeuvres that prevent conflict escalation. While reducing costs of conflict, the nonthreatening behavior exhibited by inquilines may facilitate the stability of cohabitations with hosts in the long term.

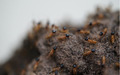

## 1 INTRODUCTION

Nature provides innumerable opportunities to observe animals coexisting (Gravel, Guichard, & Hochberg, 2011; Tokeshi, 2009), from species migrating in response to environmental conditions and temporarily interacting with local communities (e.g., Curk et al., 2020; Houghton, Doyle, Wilson, Davenport, & Hays, 2006; Kays, Crofoot, Jetz, & Wikelski, 2015) to organisms establishing long‐term interspecific relationships (Wilson, 1988). In the latter case, when animals cohabit a single place, organisms involved in the *symbiosis* (*sensu* De Bary, 1878; see Oulhen, Schulz, & Carrier, 2016) may interact continuously throughout their life span, providing excellent opportunities to investigate how species with unique independent life histories may end up sharing the same location. Not surprisingly, when choosing between locations to settle or establish a permanent housing (e.g., nests), some species may “cheat” and avoid costs with nest building, using structures built by a different organism (Kilner & Langmore, 2011). Benefits associated with nests, such as provisioning of shelter and access to resources continuously renewed over time (e.g., food and water), may, in fact, attract a variety of opportunistic organisms on the way. This “attractiveness” seems particularly common in termite nests, where an impressive diversity and abundance of non‐nestmates can be found cohabiting with original termite builders (Costa, de Carvalho, Lima‐Filho, & Brandão, 2009; Monteiro, Viana‐Junior, de Castro Solar, de Siqueira Neves, & DeSouza, 2017). Although a wide variety of species (Kistner, 1969, 1979, 1990) and trophic interactions (DeVisser, Freymann, & Schnyder, 2008) have been reported inside termite nests, here we focus on a remarkably distinct case of nest sharing between a host termite (nest builder) and a secondary opportunistic termite species, so‐called *inquiline* (sensu Araujo, 1970; nest invader).

It is worth mentioning, however, that inquilinism in termites (i.e., Isoptera) should not be mistaken with that occurring in Hymenoptera. Commonly referred as “*social parasites*” (Buschinger, 2009; Nash & Boomsma, 2008), hymenopteran inquilines (e.g., bees, wasps, ants) tend to be closely associated with hosts and exploit their social behavior intensively (Hölldobler & Wilson, 1990). In contrast, inquiline termites are thought to be primarily associated with the nest's physical structure, regardless of their associations and/or interactions with the host species (Marins et al., 2016; Shellman‐Reeve, 1997). Having proportionally smaller colonies and relatively low brood care (Korb, Buschmann, Schafberg, Liebig, & Bagnères, 2012), it seems unlikely, although possible, that inquiline termites would deplete nest resources intensively. To date, cases of inquiline termites exploiting the host's social structure to the point of compromise the host colony's fitness significantly, as occurring in inquiline ants (Buschinger, 2009), are unknown. Still, framing precisely the inquilinism observed among termite species into the spectrum of symbiotic interactions (i.e., commensalism vs. parasitism) can be challenging. Although termite–termite inquilinism has been studied under a variety of topics, such as population dynamics (Cristaldo, Rosa, Florencio, Marins, & DeSouza, 2012; Cunha, Costa, Espírito‐Santo Filho, Silva, & Brandão, 2003; DeSouza et al., 2016; Rodrigues, Costa, Cristaldo, & DeSouza, 2018), chemical communication (Cristaldo et al., 2014; Cristaldo, Rodrigues, Elliot, Araújo, & DeSouza, 2016; Jirošová et al., 2016), resource intake associations (Florencio et al., 2013), and other traits of biological significance (Campbell et al., 2016; Collins, 1980; Costa et al., 2009; Cruz et al., 2018; Darlington, 2011; Eggleton & Bignell, 1997; Redford, 1984), it remains unclear which costs (if any) inquiline termite colonies impose to their termite host species.

Regardless of how termite–termite associations should be named, it seems straightforward to recognize their relevance and advantages as models to study conflict. As typical nest builders, termites are known to respond aggressively toward invaders (Emerson, 1938; Shellman‐Reeve, 1997). Among the soldier caste of termites, for instance, aggressive behavior seems to be frequently the default response toward non‐nestmates (Noirot, 1970), with soldier individuals engaging in endless fights to protecting the colony (Binder, 1987). In addition to the soldiers’ aggression, termite workers can also take part in aggressive actions, depending on the social context (Ishikawa & Miura, 2012). With such aggressiveness in place, nests containing any species other than the builder itself (i.e., nest invaders) are likely to favor the emergence of conflict, at least in the long term. Curiously, contrary to host termites, inquiline termite colonies may be found in the wild severely depleted in their contingent of soldiers (Cunha et al., 2003). In some cases, the proportion of inquiline soldiers may account for less than one percent of the colony (e.g., *I. microcerus*; H. Hugo, personal observation). With such a reduced or virtually absent defensive caste, relying on nest invasions to persist locally may represent a considerable risk for inquilines, and an intriguing question is how cohabitation in such terms is even possible. After all, with small colonies and a few soldiers to deal with the termite host's aggression, how inquiline termite colonies manage to establish themselves in the nest? Indeed, previous studies have tackled such an issue, suggesting proximate mechanisms that would allow inquiline and host termites to meet less frequently inside the nest. It is been generally hypothesized that, immediately after successful invasions, inquilines would establish themselves in the nest by decreasing the chances of being noticed by the host termite in the first place. A known passive mechanism for inquiline termite colonies to achieve such an effect is through changes in their chemical signature, so that to become undetectable to the host species (Cristaldo et al., 2014). Alternatively, it is also possible that inquilines decrease the chance of being noticed by hosts actively, by performing behaviors that include (a) avoiding walking in galleries crowded by hosts (Mathews, 1977); (b) not conflicting with dietary requirements of the host (Florencio et al., 2013; Miura & Matsumoto, 1998); (c) intercepting hosts’ chemical signals and using the information acquired to preclude encounters with hosts (Cristaldo et al., 2014; Cristaldo, Rodrigues, et al., 2016); and (d) keeping the colony isolated from hosts by changing the nest structure (e.g., building their own galleries and sealing chambers; H. Hugo, personal observation). Despite functioning independently through different mechanisms, these behavioral strategies seem to coincide in a single outcome: By preventing proximal and direct contact, inquilines reduce the frequency of encounter with hosts.

While potentially attenuating the likelihood of conflict inside the nest, these strategies would not entirely prevent interspecific encounters from happening. For most inquilines, including the species studied here, to date there is no conclusive evidence of colonies exiting the nest after they break in, neither for nest defense nor for foraging. The only exception is the winged reproductive caste (i.e., alates) that leaves the nest during swarming to reproduce and start new colonies (Matsuura, 2010). Thus, immediately after an inquiline colony settles inside a host nest, an associated probability of interspecific encounter must be taken into account. The nest's physical boundaries confine nest occupants locally restricted and, therefore, bound to meet in the long term. Under these circumstances, inquilines would be required to behave accordingly, for instance, avoiding conflict escalation when eventually meeting nest owners. Although hinted previously (e.g., Cristaldo et al., 2014; Cristaldo, Rodrigues, et al., 2016; Florencio et al., 2013), this intuitive theoretical prediction has not been empirically tested. In fact, little is known about the dynamics of host–inquiline interactions within the nest. Knowing precisely how inquilines cope with the menace of imminent confrontation with their hosts could provide important clues about how these cohabitations hold in nature.

A conservative approach to the issue above would sustain that inquilines should replicate at the individual‐level the nonthreatening behavior they exhibit collectively, as a group. Thus, one would hypothesize that group‐level strategies to avoid conflict (highlighted above) arise, in fact, from nonthreatening postures exhibited by inquiline individuals. Here, we tested the hypothesis that, once inevitably exposed to hosts, inquiline individuals exhibit nonaggressive behaviors, displaying hence a less threatening profile. As a result, inquiline colonies would be able to collectively reduce conflict with nest owners. Under this hypothesis, we hence expected inquiline individuals to weaken conflict escalation by (a) being predominantly lethargic and minimizing encounters with hosts and (b) exhibiting low aggressiveness by avoiding either initiating or retaliating attacks from aggressors. An alternative hypothesis would be that, even though inquiline colonies behave collectively as a deceptive organism, individual termites still adopt the behavior mostly observed among termite species, that is, to engage in conflict and retaliate when eventually meeting aggressors. To test our hypothesis, we observed in detail the behavior of an obligate inquiline termite, *Inquilinitermes microcerus* Silvestri (1901) (Termitidae: Termitinae), in the presence of its host termite, *Constrictotermes cyphergaster* Silvestri 1901 (Termitidae: Nasutitermitidae). We exposed hosts and inquilines to each other under two different experimental scenarios: (a) in closed arenas that kept individuals locally restricted, thus favoring host‐inquiline encounters, and (b) in open arenas, where hosts and inquilines had the option to distance themselves from each other, by accessing an external area circumscribing the inner one.

## 2 METHODS

### 2.1 Biological model

The termite *C. cyphergaster* (hereafter, host) is a Neotropical species widely distributed in South America (Krishna, Grimaldi, Krishna, & Engel, 2013; Mathews, 1977), known to forage at night in exposed columns and without the protection of covered galleries (Moura, Vasconcellos, Araújo, & Bandeira, 2006). In this species, nests are often founded in the ground with a royal couple, but after reaching a certain size the colonies migrate to the trees, where they establish typical arboreal nests (Vasconcellos, Araújo, Moura, & Bandeira, 2007). At this phase, it is common to find colonies of *I. microcerus*, a secondary opportunistic termite species that inhabits the *C. cyphergaster* nests (hereafter, inquilines). The name *Inquilinitermes* is justified, as these termites are unable to build their own nests and are found exclusively within nests of host termites (Emerson, 1938; Mathews, 1977). Although it remains unclear how nest invasions occur, there seems to be a critical nest volume above which inquiline colonies are more likely to be found inside host nests (13.6 L, see Cristaldo et al., 2012). Indeed, the size of termite nests seems to indirectly affect inquilinism in termite species, being negatively related to the host's defense rates (DeSouza et al., 2016). Besides, while evaluating populational parameters of nests containing inquiline colonies, Rodrigues et al. (2018) reported a negative correlation between the number of host individuals and the proportion of soldier/workers. Compared to host colonies, inquiline colonies are much smaller in size, yet they are still easily detectable due to a characteristic dark lining covering their galleries (Cristaldo et al., 2012; Cunha et al., 2003; Florencio et al., 2013). Within the host nest, inquiline colonies are often associated with chambers filled with black material, previously hypothesized as waste dumped by hosts (Emerson, 1938), but still of unknown origin.

### 2.2 Study site and collection

To carry out experiments, 27 nests of the host species containing inquiline colonies were collected from two sites in the Brazilian Cerrado (Ratter, Ribeiro, & Bridgewater, 1997) located in the State of Minas Gerais (southeastern Brazil), areas classified as “equatorial savannas with dry winters” (Kottek, Grieser, Beck, Rudolf, & Rubel, 2006). From the nests collected, 15 were collected near the municipality of Sete Lagoas (19°27ʹ57ʺS, 44°14ʹ48ʺW) in July 2012, and 12 were collected near the municipality of Divinópolis (20°08ʹ20ʺS, 44°53ʹ02ʺW) in January 2015. All nests collected were transported to the laboratory and kept under suitable conditions of temperature, relative humidity, food (lichen + vegetal matter), and water availability (Collins, 1969) until the conduction of experiments. Before the experiments, all nests remained in the laboratory for 5 days maximum.

### 2.3 Defining behaviors

**FIGURE 1:**
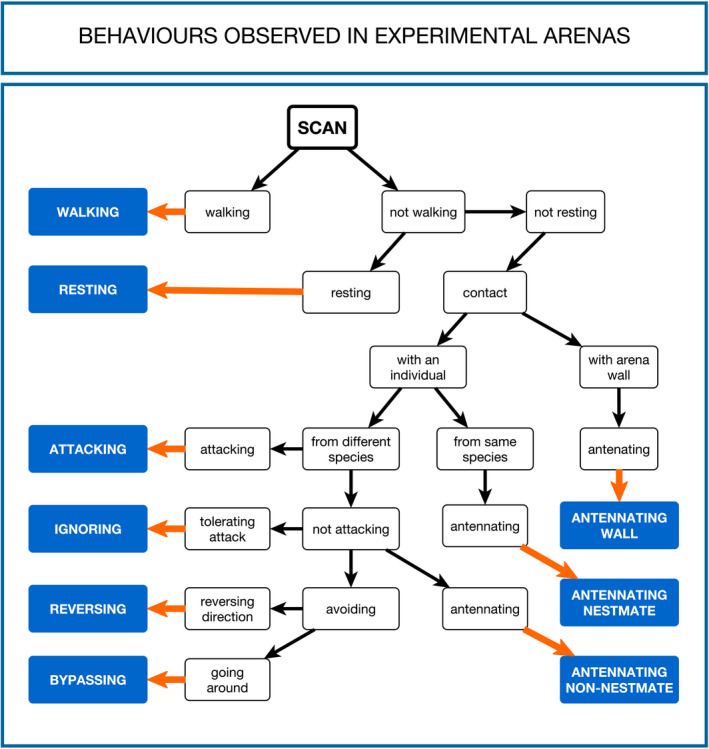
Behavioral flowchart used by observers as reference during behavior annotation. Labels were systematically organized to allow stepwise classification of observations into the nine types of behavior defined

Prior to the main experiments, we took preliminary observations to characterize behaviors possibly performed by termites. At this phase, we spent efforts to identify as many relevant behaviors as possible. All extra footage used to perform these observations (10 videos) was never used to do behavior annotation during the main experiments. Based on our preliminary observations, we designed a behavioral flowchart using simple straightforward labels systematically organized (Figure 1). Both this diagram and a list of containing brief behavioral descriptions (Table 1) were used as a reference during behavior annotation.

**TABLE 1:** Behavioral description based on preliminary observations

Behavior	Description	Classification 1	Classification 2
Resting	Focal animal remains stationary at the same place	Nonaggressive	Within‐species
Walking	Focal animal moves freely around the arena	Nonaggressive	Within‐species
Antennating wall	Focal animal reaches the arena wall and performs only antennation	Nonaggressive	Within‐species
Antennating nestmate	Focal animal encounters nestmate and performs only antennation	Nonaggressive	Within‐species
Antennating non‐nestmate	Focal animal encounters non‐nestmate and perform only antennation	Nonaggressive	Between‐species
Ignoring	Focal animal encounters non‐nestmate and do not react	Nonaggressive	Between‐species
Reversing	Focal animal encounters non‐nestmate and perform u‐turn manoeuvre	Nonaggressive	Between‐species
Passing	Focal animal encounters non‐nestmate and perform a bypass manoeuvre	Nonaggressive	Between‐species
Attacking	Focal animal encounters non‐nestmate and performs aggression	Aggressive	Between‐species

Note

We defined nine observable behaviors of relevance to our scope using ten additional video samples. For statistical analysis, we classified each behavior in two classifications: (i) within‐species versus between‐species and (ii) aggressive versus nonaggressive. Nestmates are individuals from the same species being referred, while non‐nestmates are individuals belonging to the other cohabiting species.

### 2.4 Experimental design

**FIGURE 2:**
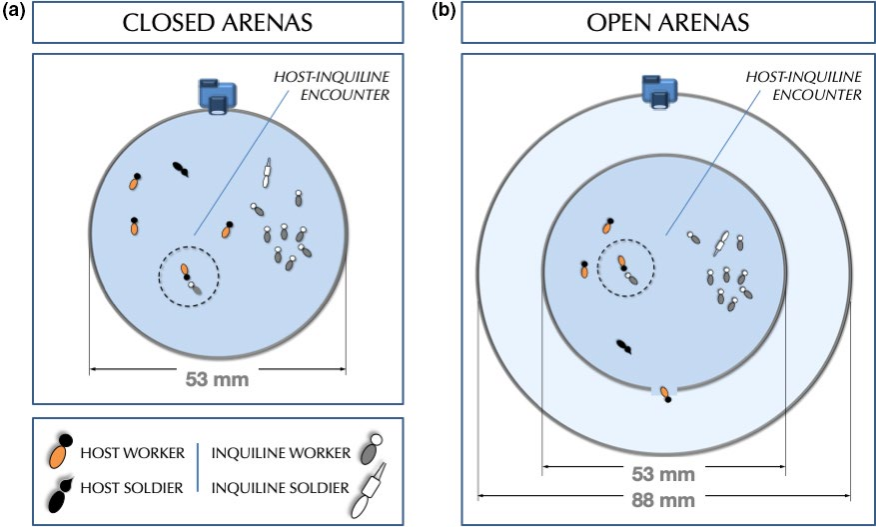
Arena settings for video recording: (a) Closed arenas and (b) open arenas. Video samples were recorded for 5 min and focal animal observation was carried out by observers using a 14” LED‐LCD 1080p screen. A gate consisted of a single opening with diameter of 3.5 mm on the arena wall connecting internal and external areas. Internal area's diameter = 53 mm. External area's diameter = 88 mm

We assessed all behavioral profiles during host–inquiline encounters as follows: Individuals of *C. cyphergaster* (hosts) and *I. microcerus* (inquilines) colonies were taken from their nests (see details in Table S2), acclimatized separately for 30 min in containers, and then gathered in experimental arenas for video recording. Because excessive desiccation compromises experiments with termites, we made efforts to prevent water loss by (a) keeping termites within their original nests before the bioassays, (b) providing a water source to the containers used to acclimatize individuals, and (c) limiting video recordings to five minutes maximum. Each arena used in bioassays consisted of a Petri dish (Ø 53 mm), cleaned with alcohol 80%, and lined at the bottom with medium flow filter paper Whatman^®^ (Grade 1:11 μm; Figure 2). Video samples were recorded using a camera Nikon D300S (1080p, 25fps) recording under the visible spectrum of light. Room temperature was set between 23°C and 24°C.

We designed two bioassays to test our predictions on the low aggressiveness of inquiline colonies with and without chance to flee. In the first bioassay (Figure 2a), individuals from both host and inquiline colonies were mutually confined in closed arenas. In the second bioassay, hosts and inquilines were gathered in arenas mostly identical to those used in the first setup, except for the presence of an exit gate. This gate consisted of a single opening (Ø 3.5 mm) on the arena wall, giving access to an external circular area (Ø 88 mm) encompassing the inner one (Ø 53 mm; Figure 2b). Using open arenas, we defined two treatments to test whether the presence of inquilines would affect host's behavior: (a) open arenas containing host and inquilines, and (b) open arenas containing only hosts. We conducted all experiments with individuals kept under density 0.12, suggested by Miramontes and DeSouza (2008) as optimal for behavioral studies with termite species. The worker‐to‐soldier ratio for each species was defined based on the caste ratio found in natural conditions, resulting hence in a proportion 4:1 for hosts and 9:1 for inquilines (for details, see Cunha et al., 2003). Also, because *I. microcerus* termites are commonly found crowded in groups inside the nest galleries (H. Hugo, personal observation), we kept the inquiline‐to‐host proportion in the arenas with inquilines outnumbering hosts locally. Thus, each experimental group contained 15 termites, being (a) one soldier and four workers for the host species and (b) one soldier and nine workers, for the inquiline species. Individuals composing a given experimental group were never present in a second bioassay, as to avoid interference from prior contact with non‐nestmates.

To capture host–inquiline interaction in all experimental arenas, we adopted focal animal sampling (Altmann, 1974) with observations taken from video samples. We recorded a total of 20 video samples of five minutes (10 using closed arenas, 10 using open arenas). Behaviors performed by host workers (HW), host soldiers (HS), inquiline workers (IW), and inquiline soldiers (IS) were annotated for each video sample using the flowchart presented in Figure 1. For each one of these categories, an individual was arbitrarily selected for focal observation (hereafter, focal animal). Thus, a total of 80 focal animals (4 castes per arena × 20 arenas) from 27 different termite nests had their behavior annotated. At total, 300 termites (15 termites per arena × 20 arenas) were placed in experimental arenas. Using a 14ʺ LED 1080p screen to watch video samples, we took three‐second observations (hereafter, scans) for each focal animal. Scans were taken at regular time intervals of 10 s, indicated to observers by scheduled sound signals. This method provided 31 scans per focal animal for each video sample, hence covering the full length of the original footage. Finally, we organized each behavioral annotation in files including all relevant information (e.g., observer, date and time of recording, room temperature) for posterior data analyses.

### 2.5 Measuring aggressiveness and host–inquiline interactivity

To measure host's and inquiline's aggressiveness and interactivity between both species in closed arenas, behaviors were classified into two classifications, as described in Table 1 (i.e., nonaggressive” versus “aggressive” in classification 1 and “within‐species” versus “between‐species” in classification 2).

### 2.6 Assessing behavioral profiles

To investigate the influence of specific individual behaviors in the general profile of hosts and inquilines, we developed a network analysis using the free software yEd Graph Editor (version 3.20). To build graphs for each caste, we performed the following procedure. Using behavioral sequences extracted from annotations, we constructed adjacent matrices containing the behavioral change for each caste (see Appendix S1, Table S3), which later was imported to yEd to draw the graphs. As typically done in standard network analysis (Brandes & Erlebach, 2005), graphs consisted of networks of nodes linked by connecting edges (i.e., directional arrows). In our case, however, nodes represented specific observable behaviors executed by focal animals, whereas connecting edges represented behavioral changes from a given behavior to another one. For instance, if individuals changed from “resting” to “walking” behavior, the behavioral change annotated would be “rest–walk.” With nine observable behaviors defined in our scope (Table 1), 81 types of behavioral change could be possibly observed. With the constructed graphs, we then calculated centrality measures (Freeman, 1978) using the number of incoming connecting edges for each node (Brandes & Erlebach, 2005). Using the calculated centrality scores, we adjusted the size of nodes to visually represent the degree of influence exerted by each behavior upon profiles, that is, the larger the size of a node, the higher its influence on the network.

### 2.7 Ethogram validation

**FIGURE 3:**
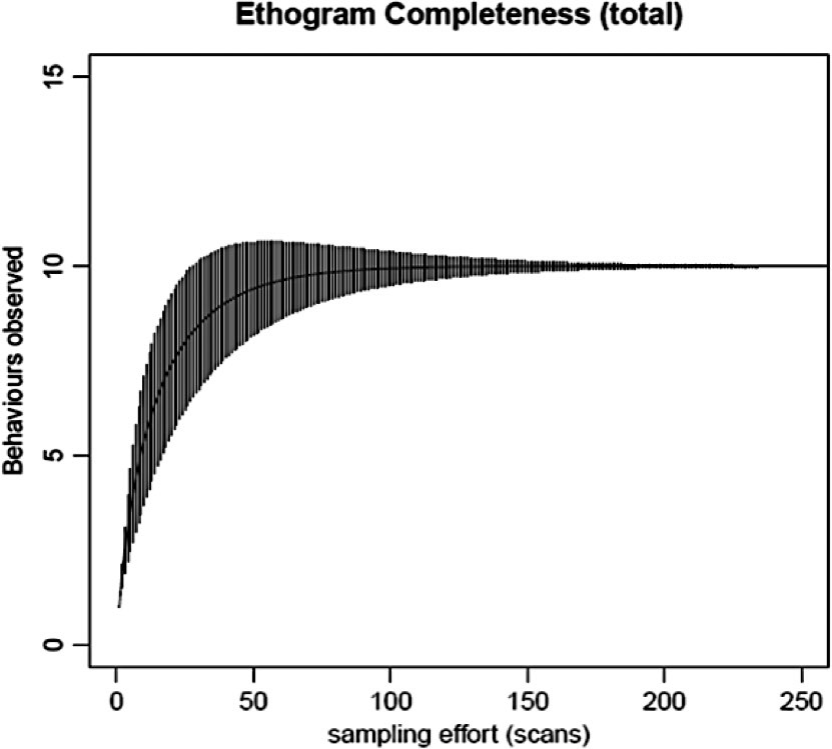
Ethogram completeness performance using behavioral accumulation curves (Dias et al., 2009): The *x*‐axis represents sampling effort, that is, the number of scans performed to observe all behaviors; the y‐axis represents the accumulative number of behaviors experimentally observed in trials

We adopted a procedure suggested by Dias, Rangel‐Negrín, Coyohua‐Fuentes, and Canales‐Espinosa (2009) to validate our ethograms, using behavioral accumulation curves (BAC) to assess an optimal balance between (a) effort with sampling and (b) ethogram completeness. A minimum of 250 independent observations would be required to efficiently capture a total of nine observable behaviors (Figure 3). In our study, we surpassed this number performing 1,240 discrete observations for the nine observable behaviors defined previously (i.e., 31 scans × 2 castes × 2 species × 10 replicates = 1,240 scans).

### 2.8 Statistical analyses

**FIGURE 4:**
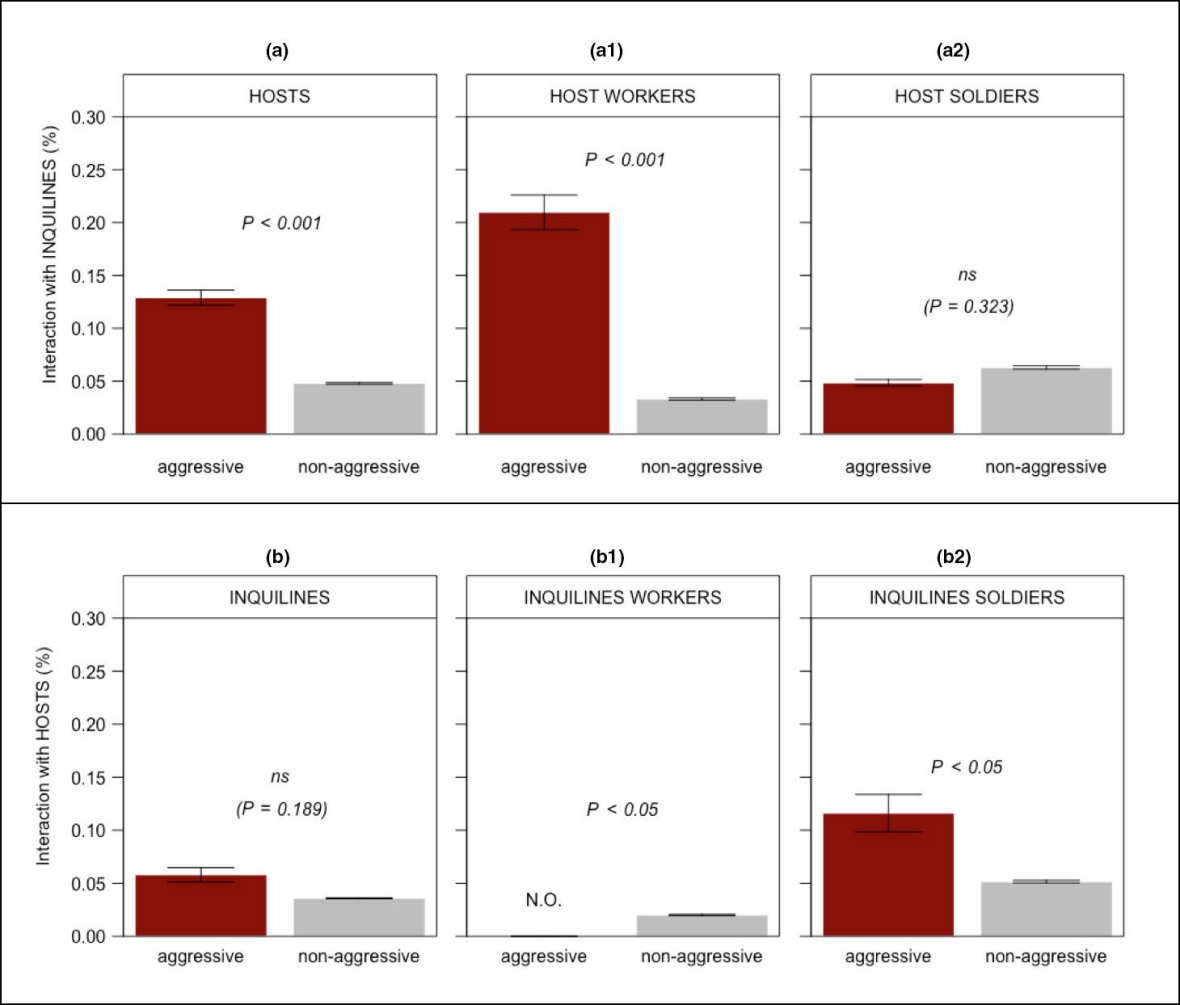
Relative interactions observed for inquilines (top row) and hosts (bottom row) in closed arenas: (a) and (b) show results for HOSTS and INQUILINES respectively, when combining worker and soldier castes into a single class; (a1) and (a2) show separate results for HOST WORKERS and HOST SOLDIERS, respectively; (b1) and (b2) show separate results for INQUILINE WORKERS and INQUILINE SOLDIERS, respectively. Proportions were individually obtained dividing the number of aggressive and nonaggressive interactions annotated by the total number of observations made. Only behaviors that occurred from an interaction are represented. *p* Values are indicated for every comparison and the bars represent *SE* (ns = nonsignificant, NO = not observed)

We performed all statistical analyses in R (R Development Core Team, 2020) using generalized linear modeling (GLM) under binomial errors with log link. For experiments in closed arenas, models included (i) the proportion of interspecific behaviors relative to all behaviors exhibited by the focal individual as the y‐var and (ii) the type of encounter (“aggressive” versus “nonaggressive”) as the x‐var. Distinct models have been tested for each of the following y‐vars: (i) behaviors exhibited by focal individuals irrespective of their caste; (ii) behaviors exhibited by focal soldiers; and (iii) behaviors exhibited by focal workers. Because the above models have been built independently for hosts and inquiline individuals, a total of six models were subjected to analysis (details are given in Figure 4). To investigate interactivity between both species, models including the x‐vars (i) behaviors between‐species and (ii) behaviors within‐species have also been tested for the y‐var (i) behavioral response exhibited by focal individuals irrespective of their caste. Relative interactions observed in closed arenas for each caste of both inquilines and hosts were obtained individually dividing the number of aggressive and nonaggressive interactions observed by the total number of observations made (31 scans per focal animal). Proportions were, hence, calculated as follows: The numerator included all instances of interactive behaviors annotated, excluding those without interaction between individuals (i.e., resting, walking, antennating wall); the denominator included the total number of observations made. Similar to the analyses conducted for closed arenas, for experiments with open arenas the models included the x‐var (i) type of assembling (“host only” versus “host with inquiline”) and the y‐var (i) mean time spent by individuals to leave the internal area. As a conservative approach, the significance of treatments was accessed as follows: First, we compared complex models to simpler ones achieved by combining treatment levels (Crawley, 2012). If the simplification did not provoke significant changes, we accepted simpler models and considered the combined treatments equivalent to each other. We then submitted adjusted models to a residual analysis, to check the suitability of the modeling equation and normality of error distribution. If required, we adjusted the error distribution by using quasi‐binomial distribution. For all tests conducted in this study, we considered an *α* = 0.05 to assess statistical significance.

## 3 RESULTS

### 3.1 Inquilines suffered attacks from hosts but responded with low aggressiveness

The proportion of aggressive interactions initiated by hosts when encountering inquilines in closed arenas was significantly higher than the proportion of nonaggressive interactions (*GLM*; *F*_1,98_ = 16.72, *p < *.001; Figure 4a). In a second analysis, considering host workers and host soldiers separately, only the former showed significant results (Figure 4a1–a2). The caste type (i.e., worker vs. soldier) was, in fact, determinant in the form of aggression inflicted by host individuals in arenas. Host workers, which were more prone to engage in conflict with aggressive interactions (Figure 4a1), physically injured inquilines, biting them in several less esclerotised portions of their softy bodies (e.g., abdomen). In contrast, being devoid of functional mechanical mandibles, host soldiers were less prone to engage in physical conflict, exhibiting instead abrupt movements with stretched antennae as an agonistic display. In the host termite *C. cyphergaster,* soldiers present in their head a snout‐like protuberance containing a frontal gland, an apparatus that contains a mixture of terpenoids often sprayed over opponents during defensive actions (Cristaldo et al., 2015). In our observations when conducting experiments, however, we were not always able to detect whether an agonistic display was followed by chemical spray, although such behavior was noticed several times in our recordings.

**FIGURE 5:**
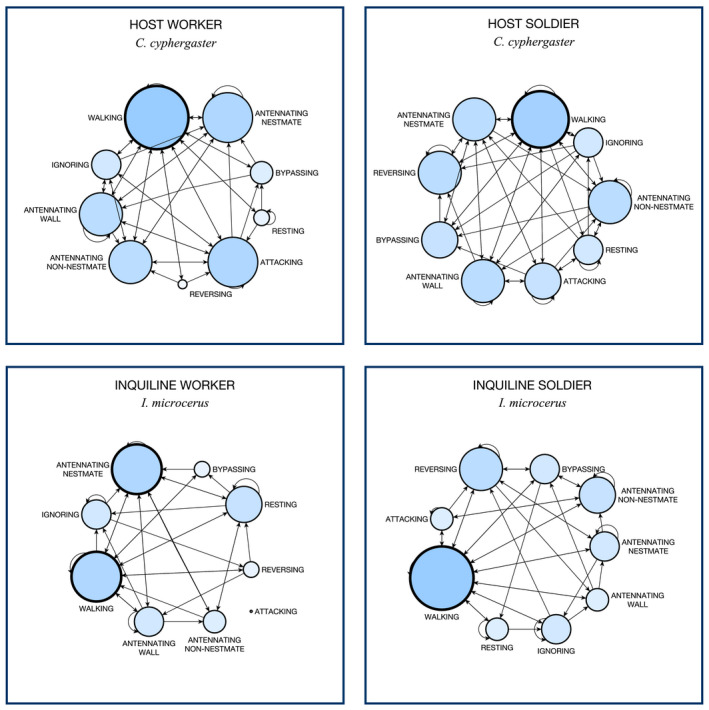
Behavioral profiles observed for each caste. Nodes represent behaviors performed by individuals, whereas connecting edges (arrows) represent behavioral changes occurred from one behavior to another. Behaviors with the highest influence on the network are highlighted with thicker node contours. Node size was adjusted using calculated centrality measures to visually represent the degree of influence exerted by each behavior upon the profiles. For inquiline worker, “attacking” was never observed and, therefore, such a behavior does not connect to the network. A version with calculated scores is provided in the Appendix S1 (Figure S1)

The proportion of aggressive interactions initiated by inquilines upon encountering hosts was not significantly higher than the proportion of nonaggressive interactions (GLM; *F*_1,98_ = 1.74, *p* = .18; Figure 4b). When threatened, or even severely injured by hosts, inquiline workers never retaliated (attacking; Figure 5). Instead, they were more likely nonaggressive (Figure 4b1), adopting evasive manoeuvres and quickly diverting away from their aggressors. These actions occurred immediately after an active contact with a host individual was established, and included behaviors characterized by avoiding the aggressor (reversing, bypassing; Figure 5). In addition to escaping from host threats, inquiline workers also performed ignoring behavior. In this case, inquiline workers actively touched by host individuals did not show any reaction to a tactile stimulus, remaining completely stationary (ignoring; Figure 5).

An exception to such a lack of aggressiveness among inquilines was the behavior of inquiline soldiers, which were more prone to engage in aggressive interactions (Figure 4b2). This aggression, however, occurred not so frequently (attacking, Figure 5) and was mostly performed in retaliation to host assaults (Appendix S1, Video S1). Also, the level of aggressive interaction observed for inquiline soldiers was less pronounced than that observed for host workers, which was the most aggressive caste among all (see Figure 4a1,b2). Aggressions performed by inquiline soldiers consisted of snapping attacks, a sudden release of slender mandibles pressed against each other producing powerful strikes over opponents (Appendix S1, Video S2).

### 3.2 Inquilines interacted little with hosts even when locally restricted

Inquilines exhibited low interactivity with host individuals, even when locally restricted and presumably more prone to meet, such as in closed arenas. The proportion of between‐species observations was significantly lower than the proportion of within‐species observations (*GLM*; *F*_1,178_ = 71.73, *p < *.001). Besides, the behavioral change of inquiline workers was characterized by a loop between resting, walking, and antennating nestmate, three behaviors without contact with the host species (Figure S2). In the Appendix S1, Table S1 contains the absolute numbers for between‐ and within‐species observations for each one of the castes.

### 3.3 Hosts were active in arenas, while inquilines were lethargic

When placed in closed arenas, host individuals performed antennation on the arena wall more frequently than inquiline individuals (GLM; *F*_1,78_ = 4.73, *p < *.005), an indication that hosts could be attempting to broaden their patrolled area. We confirmed this suspicion with the results from the second experiment, using open arenas: Host individuals quickly moved to the external area passing through the gate as soon as they found it. Either in the presence or absence of inquiline individuals, there was no difference in the mean of the time spent by host individuals to leave the internal area (19.93 ± 3.56 s, *F*_1,9_ = 0.34, *p = *.57). Inquilines, in turn, were more prone to remain stationary and never left the internal area, a result that seems to confirm the putative lethargic behavior of inquilines. A word of caution is in order regarding the open arenas bioassay: The absence of a control in which the inquiline is alone in the arena (without the host) prevents us to assert whether the inquiline is naturally lethargic, or it assumes a lethargic posture in the presence of the host. We are not in position to say, therefore, anything about the origin of such a lethargic behavior. Yet, results of the assay revealed a lethargic behavior on the part of the inquilines, and this lethargy can be interpreted as a nonaggressive behavior, regardless of its origin. In other words, our setup is indeed suitable to test whether or not the inquiline is able to flee when detecting the host: If it stays quiet, rather than fleeing or fighting, we can say that it is using a pacific strategy.

### 3.4 Inquiline's defecation prevented host aggression

We observed an unexpected response among inquiline workers: When threatened by hosts, inquiline workers deposited fecal pellets always toward the direction from which they suffered threats (Appendix S1, Video S3). Rather than usual defecations, this behavior seemed to be elicited by host aggressions as follows: When receiving attacks from backwards, individuals immediately placed fecal pellets in front of the head of aggressors and escaped forward. When threats came from any other direction, however, a different response was triggered: Before defecation, individuals first adjusted their posture to allow the fecal pellets to be dropped right in front of the aggressor's head. Only after such a move, inquiline workers defecated and escaped forward. We observed this behavior 33 times and, in all occurrences, the fecal pellet immediately prevented inquilines of being chased or receiving further attacks from aggressors. Although we did not measure whether inquiline feces have a repellent effect over hosts, it appears from our recordings that areas containing feces were less visited by host individuals. A representative example of this motion pattern is provided in the Appendix S1, Video S3.

### 3.5 Caste types showed unique behavioral profiles

We found striking differences when comparing the behavioral profile of host and inquiline termites (Figure 5). Visual representations obtained from network analyses revealed unique configurations for each one of the castes analyzed. For all caste types, walking was the behavior with the highest centrality score (walking; Figure 5), that is, with the highest influence on the network. The only caste type that presented two behaviors equally influential in the network was that of inquiline workers. In this caste type, besides walking, conspecific antennation also reached the highest centrality score (antennating nestmate; Figure 5). As highlighted above, because inquiline workers never performed an attack, this behavior presented the lowest centrally score and did not connect to the other nodes in the network (attacking; Figure 5).

## 4 DISCUSSION

It seems straightforward to understand that animals constantly surrounded by potential aggressors do not necessarily have to respond with aggression. When facing hostile interactions, engaging in conflict is only one of the behaviors that may be selected over time. Here we demonstrate that, at least for the system in the hand, nonaggressive behavior is a valid strategy, which seems to be used by inquilines (*I. microcerus*) to mitigate detrimental consequences of unexpected encounters with hosts (*C. cyphergaster*). More important, it seems to secure housing for inquiline colonies within host nests in the long term. Evasive behaviors by inquiline species have been previously suggested as one of the proximate causes of inquilinism in termites (see Cristaldo et al., 2014; Florencio et al., 2013). Here, besides providing substantial behavioral data supporting this idea, we show that once inevitably exposed to hosts, inquiline individuals exhibit nonaggressive behaviors, allowing them to display a less threatening profile and ultimately prevent conflict escalation.

### 4.1 Behavioral adaptations of a nonthreatening invader

A set of behaviors seem to support our interpretation of inquilines as nonthreatening cohabitants. First, when encountering host individuals, inquilines suffered several attacks but did not react with the same level of aggressiveness. Such a lack of aggression was markedly evident among inquiline workers: In addition to never performing a single attack during our experiments, these individuals were frequently observed moving away from their aggressors, while performing evasive manoeuvres (reversing, bypassing; Figure 5). Although the caste of inquiline soldiers was observed retaliating host attacks (attacking; Figure 5), it is important to reiterate that soldiers of *I. microcerus* are rare and often a minority in natural inquiline colonies.

A second behavior linked to the low level of aggressiveness reported (Figure 5) was the reduced mobility of inquilines compared to hosts, which per se seems to reduce host–inquiline encounters and, hence, interactions between species. In this respect, when assembled in open arenas only hosts moved to the external area over time, whereas inquilines remained idle in the inner portion. The limited mobility, however, can only be said as nonthreatening if it is not the cause for the host to move away, which was evidenced by the fact that the time spent by hosts to find the gate in open arenas was not affected by either the presence or absence of inquilines. Despite the lack of a control with only inquilines in open arenas, inquilines never completely left their initial position in our pilot experiments, remaining mostly idle wherever they were placed. One way to interpret the spatiotemporal segregation we observed would be as a direct consequence of the behavioral profile exhibited by hosts. As we have shown, hosts were much more active than inquilines in arenas, spending less time remaining stationary in the same place (Figure 5). As they walk more intensively and explore the arena more efficiently, gates would be more readily found.

A third component that seemingly affected the amount of aggression reported in arenas was defecation by inquilines. Presence of fecal pellets shortened host–inquiline contact in virtually all occasions. Consequently, host attacks toward inquilines were less frequent. This result indicates that feces may improve evasion by interrupting host aggressions. In fact, defecation as an evasive mechanism in termites is not exclusive of *I. microcerus* (e.g., in *Skatitermes*; Coaton, 1971). Such a defensive behavior may have important implications for cohabitation: If feces are sufficient to repel hosts, single pellets placed in narrowed galleries throughout the nest could prevent host contact in a very efficient inexpensive way. Besides, it is possible that while placing these pellets, *I. microcerus* would be spreading their scent throughout the entire nest, making it harder for hosts to locate the core of inquiline colonies. It is known that walls from the inquiline portion of nests may contain levels of C_12_ alcohols, a repellent for host individuals (Jirošová et al., 2016). Inside the nest, chemically mediated spatial separation of host and inquiline colonies may aid to avoid conflict.

### 4.2 The meaning of an interspecific encounter

The nonaggressive behavior observed among inquilines raises the question of whether such a strategy would be useful within the nest. After all, do encounters with the hosts represent a threat for inquiline colonies? We provide evidence that there are, at least, immediate detrimental consequences for encountering hosts. When meeting inquilines, hosts are more likely to be aggressive than nonaggressive (Figure 4), especially if the encounter includes a host worker (Figure 4a), whose functional mandibles can inflict substantial physical damage. Despite relatively less aggressive than host workers (Figure 4b), host soldiers can also attack chemically. In *C. cyphergaster*, terpenoids sprayed from the soldier's frontal gland function as an effective alarm pheromone (Cristaldo et al., 2015). Thus, once a target is sprayed, it recruits nestmates to converge upon the site and deploy themselves around it (Eisner, Kriston, & Aneshansley, 1976). In such a harsh environment, where virtually all individuals are potential aggressors, it is plausible that a nonaggressive behavior, rather than a costly aggressive profile, could be a simpler alternative solution. All in all, as compared to a belligerent set of behaviors, a nonthreatening profile would demand less elaborated actions, plausibly resulting in lower activity and reduced probability of interspecific encounter.

### 4.3 Mechanisms of conflict avoidance

Behaviors preventing conflict escalation are widespread across taxa (e.g., Akino, Knapp, Thomas, & Elmes, 1999; Aureli, Cords, & van Schaik, 2002; Baan, Bergmüller, Smith, & Molnar, 2014; Gobush & Wasser, 2009; Lhomme, Ayasse, Valterová, Lecocq, & Rasmont, 2012; Nehring, Dani, Turillazzi, Boomsma, & d'Ettorre, 2015; Pierce et al., 2002; Thierry et al., 2008). In termites specifically, aggressiveness depends on a range of ecological factors such as diet (Florane, Bland, Husseneder, & Raina, 2004), caste ratios (Roisin, Everaerts, Pasteels, & Bonnard, 1990), nestmate recognition (Delphia, Copren, & Haverty, 2003; Haverty & Thorne, 1989), group composition (Haverty & Thorne, 1989), territoriality (Adams & Levings, 1987; Ferreira et al., 2018; Levings & Adams, 1984), and resource availability (Cristaldo, Araújo, et al., 2016). In addition, aggression between termite colonies of the same species, which would be presumably less predictable due to a higher relatedness, may be inconsistent (Binder, 1987). Due to a behavioral plasticity (Ishikawa & Miura, 2012), species may respond aggressively in some cases (Su & Haverty, 1991) and lack aggression in others (Delaplane, 1991; Neoh, Indiran, Lenz, & Lee, 2012). These reports together indicate that, across taxa, certain species may exhibit a much lower level of aggressiveness, as compared to other organisms in the group which they belong.

The symbiosis between *C. cyphergaster* and *I. microcerus* is a representative case of obligatory inquilinism in termites, which means that, at least for inquiline species, nest sharing has become mandatory (Shellman‐Reeve, 1997). The evolutionary costs and drawbacks of such specialization by inquilines remain to be assessed, although the benefits associated with nest invasion seem to be straightforward: Nest invaders are not required to spend time and energy building their own home. At the same time, being nest building a demanding, costly process (Korb & Linsenmair, 1999; Stuart, 1967), one would expect such inquiline invasions to be not strictly in the interest of hosts. In this sense, it would be reasonable to think of a scenario in which hosts would endeavor to detect inquilines, whereas inquilines would try to go unnoticed by hosts. This evasive behavior, consistently exhibited at the individual level, confers a conflict avoiding strategy to the inquiline colony as a whole. Under the effect of such driving forces, it is likely that an evolutionary arms race between species would take place (Dawkins & Krebs, 1979), leading hosts and inquilines to reach well‐adjusted behavioral profiles. In doing so, each species would become highly specialized in dealing with its cohabiting neighbor (Kilner & Langmore, 2011).

### 4.4 Cohabitation and conflict

Our findings support a notion that hostile interactions do not necessarily lead to increased aggressiveness between opponents, especially if asymmetric aggression or lack of reciprocal retaliation is in place. Although being common in nature, conflict between species can be a limiting factor for coexistence. Excessively high levels of aggression tend to jeopardize relationships between organisms, surpassing acceptable thresholds and leading entire colonies to collapse. The behavioral adaptations we describe seem to allow inquiline colonies to manage the amount of aggression received from their hosts. Such a nonthreatening individual behavior may play a fundamental role in cohabitation, as it seems to increase considerably the chances of a stable (although asymmetric) relationship between host and inquiline colonies. Further research should explore the contributions of such individual actions on the collective patterns observed for the system. While in line with previous reports on cohabitation between termite societies, our findings reinforce the growing view of conflict management as a critical component of socially complex systems. Finally, descriptions of other similar systems, in which recipients of aggression are subjected to locally restricted hostile environments, should contribute to putting conflict and its consequences in a broader perspective, adding novel insights for studies involving multiple group‐living organisms.

## CONFLICT OF INTEREST

The authors declare that there is no conflict of interest.

## AUTHOR CONTRIBUTION

**Helder Hugo:** Data curation (equal); Formal analysis (equal); Funding acquisition (equal); Investigation (equal); Methodology (equal); Project administration (equal); Visualization (lead); Writing‐original draft (lead); Writing‐review & editing (equal). **Paulo F. Cristaldo:** Data curation (equal); Formal analysis (equal); Funding acquisition (equal); Investigation (equal); Methodology (equal); Writing‐review & editing (equal). **Og DeSouza:** Conceptualization (equal); Data curation (equal); Formal analysis (equal); Funding acquisition (equal); Investigation (supporting); Project administration (lead); Supervision (lead); Validation (equal); Visualization (equal); Writing‐review & editing (equal).

## ETHICAL STATEMENT

All permits required for the present study were obtained, complying with relevant regulations governing animal research in Brazil. This includes (a) permits from the Brazilian Institute for the Environment and Renewable Natural Resources (IBAMA, no. 33094); (b) permission from the Brazilian Enterprise for Agricultural Research (EMBRAPA) at Sete Lagoas; (c) permission from landowners at the Divinópolis site to conduct the study on their property; and (d) tacit approval from the Brazilian Federal Government implied by employing authors to conduct scientific research. None of the sampled species had protected status. No genetic information was accessed in the study.

### OPEN RESEARCH BADGES

This article has earned an Open Data Badge for making publicly available the digitally‐shareable data necessary to reproduce the reported results. The data is available at https://doi.org/10.5061/dryad.r2280gb98.

## Supporting information

Appendix S1

Video S1

Video S2

Video S3

## Data Availability

In compliance with the *Ecology and Evolution's* policy on data archiving, the dataset supporting the results of this paper is archived and publicly available in the Dryad Digital Repository, accessible in the following link: https://doi.org/10.5061/dryad.r2280gb98.
