# Supplementary material for: Nonaggressive behavior: A strategy employed by an obligate nest invader to avoid conflict with its host species
Source: Ecol Evol. 2020 Jul 24;10(16):8741–54. doi: 10.1002/ece3.6572 (PMC7452783; doi:10.1002/ece3.6572)
Supplement: Supplementary file 1 — Appendix S1 [file ECE3-10-8741-s001.pdf]

APPENDIX

Table S1. Absolute numbers and percentage of Between-species and Within-species observations taken from video-samples in closed arenas. Data is presented for hosts (*C. cyphergaster*) and inquilines (*I. microcerus*), and their respective castes.

Group observed	Number of focal animals (n)	Observations	
		Between-species	Within-species
**HOSTS (*C. cyphergaster*)**	**10**	**199 (32%)**	**421 (68%)**
Host workers	5	106 (34%)	204 (66%)
Host soldiers	5	93 (30%)	217 (70%)
**INQUILINES (*I. microcerus*)**	**10**	**125 (20%)**	**495 (80%)**
Inquiline workers	5	25 (8%)	285 (92%)
Inquiline soldiers	5	100 (32%)	210 (68%)
**TOTAL**	**20**	**324 (26%)**	**916 (74%)**

Table S2. Inquiline and host colonies used in each trial recorded for behavioural observation. “Nest1” and “Nest2” refers to the nests from which “Species1” and “Species2” were collected from, respectively.

**CLOSED ARENAS - Focal animal sampling - *Constrictotermes cyphergaster & Inquilinitermes microcerus***						
RecordID	Assemble type	Colonies collected from	Colony1 (worker-soldier ratio)	Nest1	Colony2 (worker-soldier ratio)	Nest2
5	Hosts vs. Inquilines	one nest (N13)	*C. cyphergaster* (4w1s)	N13-2012	*I. microcerus* (9w1s)	N13-2012
8	Hosts vs. Inquilines	one nest (N12)	*C. cyphergaster* (4w1s)	N12-2012	*I. microcerus* (9w1s)	N12-2012
18	Hosts vs. Inquilines	one nest (N19)	*C. cyphergaster* (4w1s)	N19-2012	*I. microcerus* (9w1s)	N19-2012
48	Hosts vs. Inquilines	one nest (N30)	*C. cyphergaster* (4w1s)	N30-2012	*I. microcerus* (9w1s)	N30-2012
66	Hosts vs. Inquilines	one nest (N33)	*C. cyphergaster* (4w1s)	N33-2012	*I. microcerus* (9w1s)	N33-2012
12	Hosts vs. Inquilines	two nests (N15 & N11)	*C. cyphergaster* (4w1s)	N15-2012	*I. microcerus* (9w1s)	N11-2012
23	Hosts vs. Inquilines	two nests (N20 & N18)	*C. cyphergaster* (4w1s)	N20-2012	*I. microcerus* (9w1s)	N18-2012
36	Hosts vs. Inquilines	two nests (N22 & N27)	*C. cyphergaster* (4w1s)	N22-2012	*I. microcerus* (9w1s)	N27-2012
61	Hosts vs. Inquilines	two nests (N32 & N28)	*C. cyphergaster* (4w1s)	N32-2012	*I. microcerus* (9w1s)	N28-2012
73	Hosts vs. Inquilines	two nests (N24 & N38)	*C. cyphergaster* (4w1s)	N24-2012	*I. microcerus* (9w1s)	N38-2012
**OPEN ARENAS - Focal animal sampling - Constrictotermes cyphergaster & Inquilinitermes microcerus**						
RecordID	Assemble type	Colonies collected from	Colony1 (worker-soldier ratio)	Nest1	Colony2 (worker-soldier ratio)	Nest2
209	Hosts vs. Inquilines	one nest (N209)	*C. cyphergaster* (4w1s)	N209-2015	*I. microcerus* (9w1s)	N209-2012
210	Hosts vs. Inquilines	one nest (N210)	*C. cyphergaster* (4w1s)	N210-2015	*I. microcerus* (9w1s)	N210-2012
211	Hosts vs. Inquilines	one nest (N211)	*C. cyphergaster* (4w1s)	N211-2015	*I. microcerus* (9w1s)	N211-2012
214	Hosts vs. Inquilines	one nest (N214)	*C. cyphergaster* (4w1s)	N214-2015	*I. microcerus* (9w1s)	N214-2012
216	Hosts vs. Inquilines	one nest (N216)	*C. cyphergaster* (4w1s)	N216-2015	*I. microcerus* (9w1s)	N216-2012
208	Only hosts (control)	one nest (N208)	*C. cyphergaster* (4w1s)	N208-2015	no inquilines used	n.a.
212	Only hosts (control)	one nest (N212)	*C. cyphergaster* (4w1s)	N212-2015	no inquilines used	n.a.
213	Only hosts (control)	one nest (N213)	*C. cyphergaster* (4w1s)	N213-2015	no inquilines used	n.a.
217	Only hosts (control)	one nest (N217)	*C. cyphergaster* (4w1s)	N217-2015	no inquilines used	n.a.
218	Only hosts (control)	one nest (N218)	*C. cyphergaster* (4w1s)	N218-2015	no inquilines used	n.a.

Table S3 Adjacency matrices containing the behavioural change for each caste of hosts (*C. cyphergaster*) and inquiline (*I. microcerus*). The data was used to extract centrality measures and draw the networks in yED (Abbreviations: walk=walking; rest=resting; aW=antennating wall; aCS=antennating nestmate; aHS=antennating non-nestmate; ig=ignoring; pa=bypassing; rever=reversing; att=attacking).

Adjacency matrix - Constrictotermes cyphergaster (worker)									
	aCS	aHS	aW	att	ig	pa	rest	rever	walk
aCS	4	1	3	0	0	0	0	0	8
aHS	1	0	0	3	1	0	0	0	3
aW	3	1	16	2	1	0	0	0	3
att	3	1	3	11	1	1	0	0	6
ig	1	1	1	1	0	0	0	0	2
pa	3	0	1	0	0	0	0	0	2
rest	0	0	0	1	0	1	1	0	2
rever	0	1	0	1	0	0	0	0	1
walk	1	3	3	9	3	3	3	3	26

Adjacency matrix - Constrictotermes cyphergaster (soldier)									
	aCS	aHS	aW	att	ig	pa	rest	rever	walk
aCS	2	1	4	1	0	1	0	1	7
aHS	0	2	1	0	0	1	2	0	3
aW	2	0	6	2	3	0	0	1	1
att	2	1	1	1	0	1	1	0	2
ig	0	2	1	0	2	1	0	1	4
pa	1	0	0	0	0	0	0	2	3
rest	2	1	0	1	2	0	15	0	0
rever	1	0	0	0	0	0	0	1	7
walk	6	4	3	4	4	2	3	3	24

Adjacency matrix - Inquilinitermes microcerus (worker)									
	aCS	aHS	aW	ig	pa	rest	rever	walk	att
aCS	11	1	3	0	0	9	0	6	0
aHS	1	0	0	0	0	1	0	1	0
aW	1	1	2	1	0	0	0	2	0
ig	1	0	0	2	0	0	1	0	0
pa	1	0	0	0	0	0	0	2	0
rest	7	1	0	2	1	24	0	11	0
rever	0	0	1	0	0	3	0	1	0
walk	8	0	1	1	2	12	3	25	0
att	0	0	0	0	0	0	0	0	0

Adjacency matrix - Inquilinitermes microcerus (soldier)									
	aCS	aHS	aW	att	ig	pa	rest	rever	walk
aCS	5	2	0	0	1	0	0	1	10
aHS	0	1	0	2	0	1	0	0	3
aW	1	0	0	0	0	1	0	0	2
att	0	1	0	12	0	0	0	2	5
ig	0	0	1	0	1	0	0	1	4
pa	0	1	0	0	0	0	2	2	4
rest	0	0	0	0	1	0	4	0	3
rever	2	0	2	0	0	3	0	2	5
walk	11	2	1	6	4	4	1	6	27

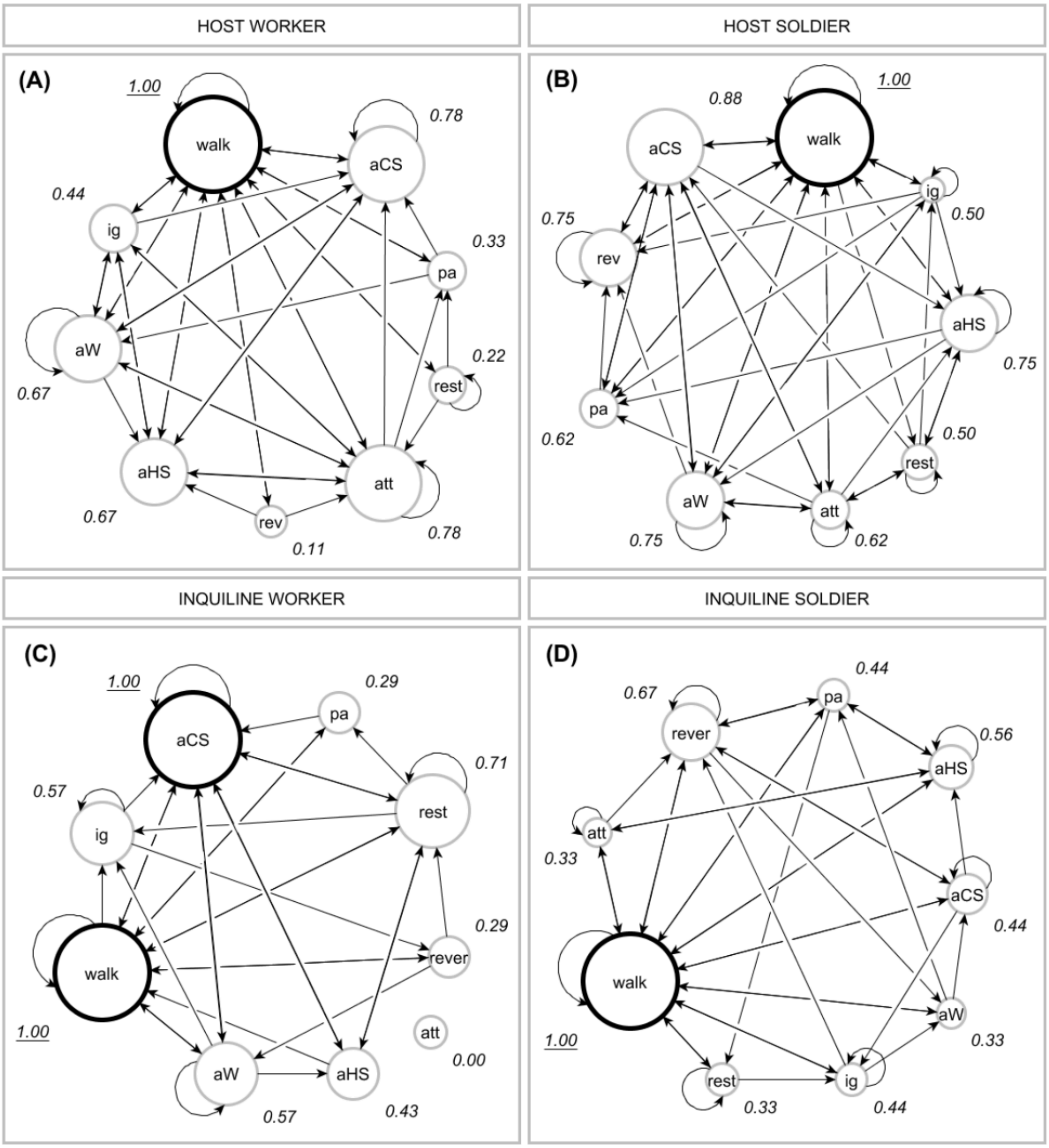

Figure S1. Behavioural profiles observed for each caste (with scores of centrality measure). Nodes represent behaviours performed by individuals, whereas connecting edges (arrows) represent behavioural changes occurred from one behaviour to another. Behaviours with the highest influence on the network are highlighted with thicker node contours. Node size was adjusted using calculated centrality measures (scores) to visually represent the degree of influence exerted by each behaviour upon the profiles. (Abbreviations: walk=walking; rest=resting; aW=antennating wall; aCS=antennating nestmate; aHS=antennating non-nestmate; ig=ignoring; pa=bypassing; rever=reversing; att=attacking).

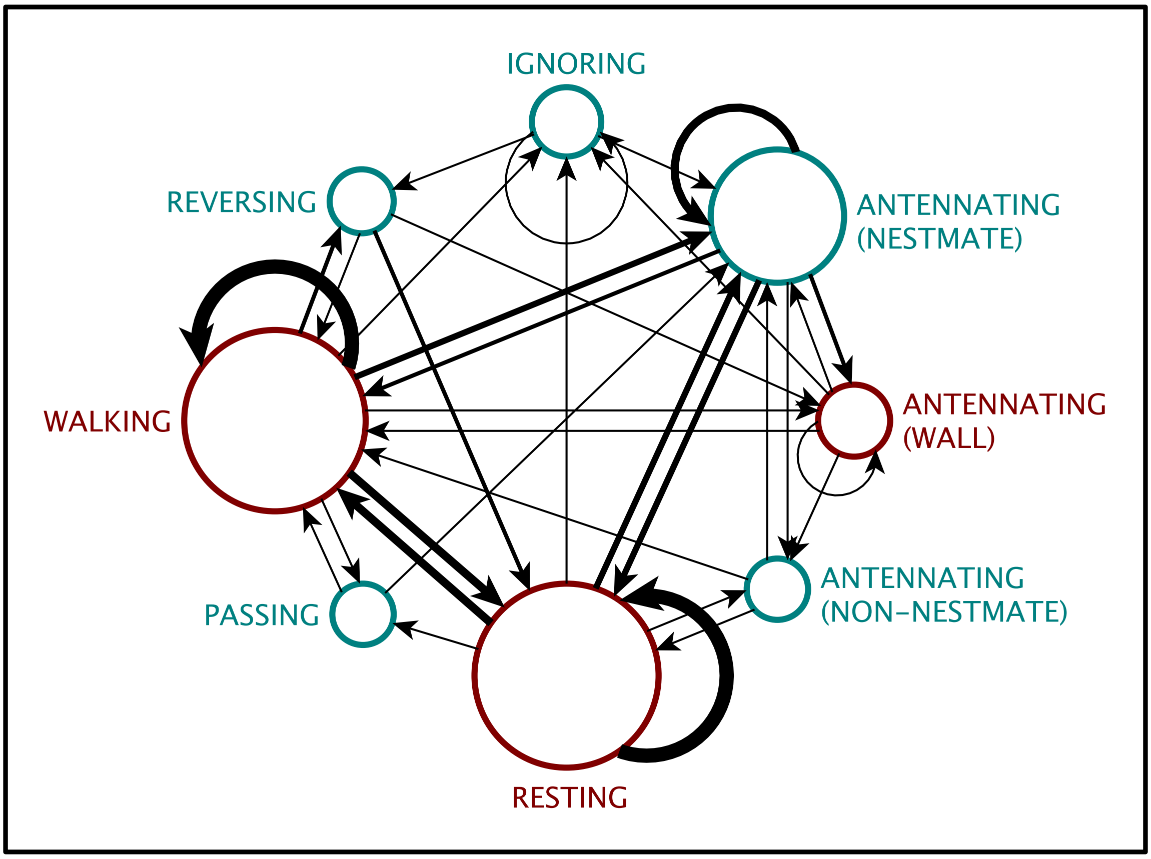

Figure S2. Behavioural change for inquiline workers: behaviours performed by individuals are represented by nodes, whereas connecting edges (arrows) represent behavioural changes occurred from one behaviour to another. Node size was adjusted using behavioural frequencies extracted from the annotation. The width of edges indicate how frequently a given behavioural change occurred.

Video S1

S1_snapping.mov

This video was recorded in lab conditions with a fixed individual of *Termes* spp. It shows the mechanism of snapping, also present in *I. microcerus* and other termite species with soldiers provided of slender mandibles.

Video S2

S2_snapping2.mov

This video was recorded in lab conditions with a free individual of *I. microcerus* in the presence of *C. cyphergaster*. It shows snapping events performed by the inquiline soldier of in retaliation to host threats.

Video S3

S3_defecation.mov

This video was recorded in lab conditions with individuals of *I. microcerus* and *C. cyphergaster* in experimental arenas. It shows the aggressive nature of host-inquiline encounters and some of the evasive behaviours performed by inquilines as response. Markers were included to highlight when aggressive interactions happened. The defensive mechanism using defecation is the depicted in the footage with several events
